# Symptomatic Cerebral Vasospasm After Clipping of an Unruptured Intracranial Aneurysm: A Case Report and Literature Review

**DOI:** 10.7759/cureus.90339

**Published:** 2025-08-17

**Authors:** Maoki Matsubara, Yoshiki Takizawa, Yuzo Saito, Yoshinobu Sekihara, Norihiro Ishii

**Affiliations:** 1 Neurological Surgery, New Tokyo Hospital, Matsudo, JPN

**Keywords:** cerebral vasospasm, delayed ischemic neurological deficit, intracranial aneurysm growth, microsurgical aneurysm clipping, middle cerebral artery infarction

## Abstract

Symptomatic cerebral vasospasm after clipping of unruptured intracranial aneurysms (UIA) is extremely rare. Furthermore, its pathophysiology and risk factors are poorly understood. Delayed diagnosis and treatment can lead to severe neurological deficits. We report the case of a 53-year-old woman who developed delayed symptomatic cerebral vasospasm seven days after clipping of an unruptured left middle cerebral artery (MCA) aneurysm. The patient presented with right-sided hemiparesis and motor aphasia. Digital subtraction angiography revealed vasospasms from the M1 to M2 segments. Treatment with intra-arterial fasudil injection and continuous intravenous fasudil with ozagrel sodium resulted in complete neurological recovery. Cerebral vasospasm after UIA clipping is uncommon and may be overlooked, thereby resulting in serious sequelae.

## Introduction

Cerebral vasospasm is a well-recognized complication following subarachnoid hemorrhage (SAH) from ruptured cerebral aneurysms, typically occurring between the sixth and eighth days after rupture [1]. In contrast, symptomatic cerebral vasospasm after clipping of an unruptured intracranial aneurysm (UIA) is extremely rare, with only a limited number of case reports available in the literature [1-15]. The pathophysiology of vasospasm in UIA remains poorly understood, and the absence of subarachnoid blood makes early diagnosis challenging. Furthermore, the risk factors, optimal treatment strategies, and long-term outcomes are not well established, owing to the rarity of this condition. Delayed diagnosis and treatment can lead to permanent neurological deficits, underscoring the importance of neurosurgeons' awareness of this complication.

We present a case of delayed symptomatic cerebral vasospasm following clipping of an unruptured middle cerebral artery (MCA) aneurysm, along with a comprehensive literature review of similar cases.

## Case presentation

**Figure 1:**
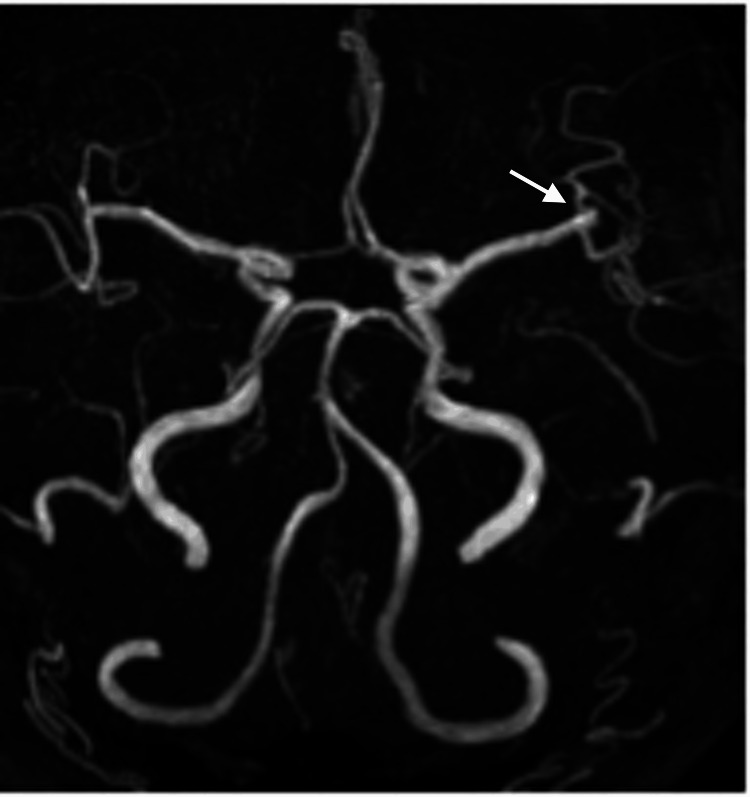
Preoperative magnetic resonance imaging A 3.8 mm-diameter unruptured saccular aneurysm (white arrow) at the bifurcation of the left middle cerebral artery (MCA).

A 53-year-old woman was diagnosed with a left MCA aneurysm based on a magnetic resonance imaging (MRI) conducted two years ago for the evaluation of headaches. Subsequent follow-up imaging indicated an increase in the size of the aneurysm, leading her to seek treatment. The patient had no history of smoking, alcohol consumption, family history of cerebrovascular disease, medications, allergies, or comorbidities. Preoperative MRI revealed a left MCA bifurcation aneurysm measuring 3.8 mm × 1.7 mm (Figure 1), and the patient was neurologically intact, with no focal deficits.

**Figure 2:**
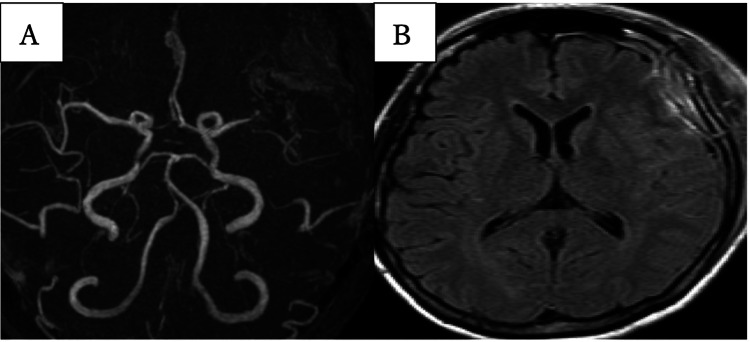
MRA and FLAIR image on postoperative day 7 (A) Poor visualization of the left MCA; (B) Signal changes in the left MCA lesion MRA, magnetic resonance angiography; FLAIR, fluid-attenuated inversion recovery; MCA, middle cerebral artery

The patient underwent a left frontotemporal craniotomy with microsurgical clipping of the aneurysm. Intraoperative findings revealed no evidence of SAH or blood products around the aneurysm, and no temporary clips were used during the procedure. Additionally, the proximal portion of the MCA was not manipulated. The aneurysm was clipped successfully without complications. Computed tomography performed on the same day showed no abnormalities or SAH. The patient had an uneventful initial postoperative course until postoperative day 7, when she developed acute-onset right-sided hemiparesis and motor aphasia. Magnetic resonance angiography (MRA) revealed poor visualization of the left MCA, and fluid-attenuated inversion recovery (FLAIR) revealed slight signal changes in the same lesion (Figure 2). Considering the possibility of symptomatic seizures and cerebral ischemia, treatment was initiated with levetiracetam to prevent seizures, dextran for hemodilution, and cilostazol for antiplatelet therapy and vasodilation.

**Figure 3:**
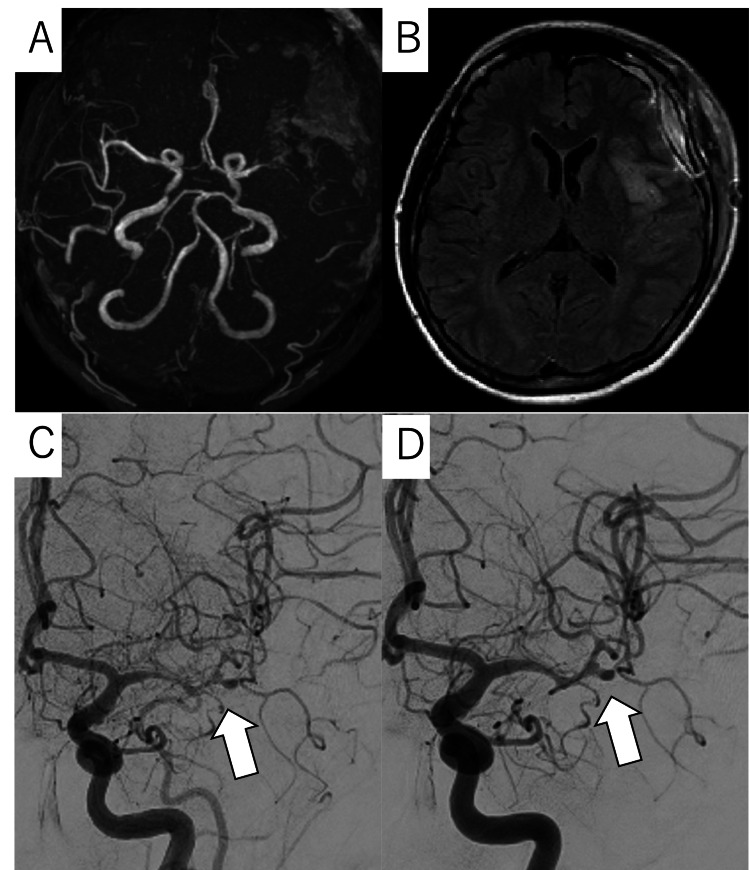
MRA, FLAIR, and DSA images on postoperative day 9 (A) Poor visualization of the left MCA; (B) Clarification and enlargement of ischemic lesions; (C) DSA before treatment revealed vasospasm in the left MCA territory (arrow); (D) DSA following intra-arterial fasudil injection showed improvement in the vasospasm (arrow) MRA, magnetic resonance angiography; FLAIR, fluid-attenuated inversion recovery; DSA, digital subtraction angiography; MCA, middle cerebral artery

On postoperative day 9, the patient’s neurological symptoms worsened, and MRI showed new ischemic lesions (Figures 3A-3B). Emergency digital subtraction angiography revealed severe vasospasm affecting the M1-M2 segments of the left MCA (Figure 3C). As an additional treatment, intra-arterial fasudil injection was performed, resulting in immediate angiographic improvement of vasospasm (Figure 3D). In addition to the previous treatment, continuous intravenous administration of fasudil and ozagrel sodium was initiated, and the patient showed gradual neurological improvement. Follow-up angiography on postoperative day 17 demonstrated mild residual vasospasm; therefore, an intra-arterial fasudil injection was administered, which again resulted in improvement. By postoperative day 29, the patient had complete neurological recovery, with a National Institutes of Health Stroke Scale score of 0 and a modified Rankin Scale score of 0. She was discharged without any neurological deficit. Afterward, she did not show recurrence or any unusual events during a one-year follow-up.

## Discussion

Although symptomatic vasospasm with aneurysmal SAH is well-recognized, there is limited literature regarding symptomatic vasospasm after UIA clipping [1-15]. This condition is difficult to predict and can lead to clinically significant neurological deficits, owing to delayed treatment [5,9].

The development of vasospasms is thought to result from the interaction between various substances in the blood, including oxyhemoglobin, endothelin, and nitric oxide [3,7]. It is generally accepted that the risk of vasospasm increases with the thickness of the subarachnoid hematoma. However, the mechanism underlying vasospasm without hematoma remains unknown. Several hypotheses have been proposed to explain this phenomenon, including intraoperative SAH, mechanical stimulation (use of multiple clips or temporary clips), hypothalamic dysfunction, transient vasculitis caused by metal allergy, and involvement of the trigeminal-cerebrovascular system [3-5,8]. Recently, the involvement of interleukin-6 (IL-6) in vasospasms after SAH has been increasingly reported [16,17]. According to Osuka et al., IL-6 levels in the cerebrospinal fluid (CSF) are elevated after SAH, thereby contributing to the development of cerebral vasospasms [16]. Moreover, IL-6 is thought to exhibit similar changes not only after SAH, but also in response to surgical stress and traumatic injury [18]. These findings suggest that inflammatory cytokines, such as IL-6, may play a crucial role in the development of vasospasm, even in the absence of SAH. However, the underlying mechanism remains unclear.

To better understand this phenomenon, we conducted a comprehensive literature review and identified 21 cases of symptomatic cerebral vasospasm following UIA clipping, including the present case (Table 1). Analysis of the demographic and clinical characteristics revealed a mean age of 57 years (range, 21-72 years) with a strong female predominance (18 cases, 86%). The left MCA was the most affected (nine cases), followed by the right MCA (four cases) and the anterior communicating artery (three cases). The mean aneurysm size was 5 mm (range, 3.7-10 mm), and temporary clips were used in eight of the 15 cases for which data were available. The mean onset was nine days postoperatively (range, 1-29 days), with postoperative complications including intracerebral hemorrhage (one case), subdural hematoma (two cases), and infarction (one case). Regarding neurological outcomes, 12 cases (57%) had residual deficits, with higher rates observed in females (11 cases, 61%) compared to males (one case, 33%). Concerning the location of onset, there were eight cases after discharge and 10 cases during hospitalization; however, there was no significant association with the presence of sequelae (five out of eight cases, 63% vs. six out of 10 cases, 60%).

**Table 1:** Reports on cerebral vasospasm after clipping of unruptured intracranial aneurysms M, male; F female; Rt, right; Lt, left; MCA, middle cerebral artery; Opth; ophthalmic artery; ICAtop; top of the internal carotid artery; Acom, anterior communicating artery; IC-PC, internal carotid-posterior communicating artery; PCom, posterior communicating artery; EDH, epidural hemorrhage; ICH, intracerebral hemorrhage; POD, postoperative day; tripleH, induced hypertension, hypervolemia, hemodilution

Author	Age	Sex	Location	Size	Temporary clip	Note	Symptoms	POD	Occurrence place	Treatment	Deficit
Bloomfield and Sonntag (1985) [1]	54	F	Rt. MCA	7	Unknown	Intraoperative spasm	Hemiparesis	9	Home	Dexamethasone	Hemiparesis
Gutiérrez et al. (2001) [2]	55	F	Lt. Opth	5	Unknown	-	Aphasia, Hemiparesis, loss of consciousness	1	Hospital	Intraarterial papaverine	Hemiparesis
Kitazawa et al. (2005) [3]	21	F	Lt. Paraclinoid	4	+	-	Aphasia	12	Hospital	Angioplasty + tripleH	None
Kitazawa et al. (2005) [3]	63	F	Lt. Paraclinoid	4	+	Postoperative EDH	Aphasia, Hemiparesis	9	Unknown	TripleH	None
Paolini et al. (2005) [4]	47	F	Rt. MCA	8	+	Postoperative ICH	Aphasia, Hemiparesis	28	Home	Hydration + Antithrombotics	Dysphasia
Yang et al. (2015) [5]	41	F	Lt. ICAtop	5	+	-	Aphasia, Hemiparesis	28	Home	Intraarterial nicardipine + Hydration + Antithrombotics	Dysphasia
Yang et al. (2015) [5]	61	F	Lt. MCA	6	+	-	Aphasia	10	Unknown	Intraarterial papaverine + Hydration + Antithrombotics	Dysphasia
Tsyben et al. (2016) [6]	53	F	Lt. MCA	5	-	-	Hemiparesis	2	Hospital	Intraarterial verapamil	Hemiparesis, Dysphasia
Tsyben et al. (2016) [6]	70	M	Acom, Lt. MCA	Unknown	-	-	Hemiparesis	2	Hospital	Intraarterial verapamil	None
Hashimoto et al. (2016) [7]	62	F	Lt. IC-PC	5	-	-	Aphasia, Hemiparesis	11	Hospital	Hydration + Antithrombotics	Acalculia, Dysphasia
Campe et al. (2019) [8]	69	F	Rt. MCA	Unknown	-	Preoperative headache	Hemiparesis	12	Home	Antithrombotics + nimodipine	None
Cuoco et al. (2020) [9]	53	F	Lt. MCA	5	+	Recurrent spasm of right PCA on POD26	Hemiparesis, Hemianopsia	13	Home	Intraarterial verapamil	Hemianopsia
Anania et al. (2020) [10]	59	F	Lt. MCA	5	+	Lt frontal hypodensity	Aphasia, Hemiparesis	6	Hospital	Intraarterial nimodipine	Hemiparesis, Dysphasia
Knight et al. (2020) [11]	68	M	Acom	5	Unknown	Contralateral side vasospasm	Aphasia	5	Home	Intraarterial nicardipine + tripleH	Short-term memory deficit
Vachata et al. (2020) [12]	65	F	Lt. MCA	10	-	-	Aphasia	5	Hospital	TripleH + milrinone + nimodipine	None
Vachata et al. (2020) [12]	72	M	Lt. MCA	7	-	-	Aphasia	6	Home	TripleH + milrinone + nimodipine	None
Kim et al. (2020)[13]	51	F	Acom	5	+	Intraoperative rupture	Dysphasia	14	Home	Intraarterial nimodipine	None
Kim et al. (2020)[13]	45	F	Lt. Pcom	Unknown	Unknown	-	Aphasia, Hemiparesis	12	Unknown	Intraarterial nimodipine	None
Peterson et al. (2020) [14]	67	F	Rt. MCA	8	Unknown	-	Hemiparesis	29	Hospital	Intraarterial verapamil + tripleH + aspirin	Hemiparesis
Kim et al. (2024) [15]	Middle aged	F	Lt. MCA	3.7	Unknown	Postoperative EDH+	Aphasia, Hemiparesis	7	Other hospital	Intraarterial nimodipine	Dysphasia
Our case	53	F	Lt. MCA	3.8	-	-	Aphasia, Hemiparesis	7	Hospital	Intraarterial fasudil	None

These findings suggest several important patterns. First, in left MCA aneurysms, there is a relatively high number of reports associated with vasospasm after UIA clipping. Secondly, vasospasm after UIA clipping is observed more frequently in women and with greater severity compared to men. Thirdly, compared with SAH-associated vasospasm, the onset of vasospasm after UIA clipping typically has a delayed onset and tends to result in more severe outcomes. The high prevalence of vasospasms in left MCA aneurysms is likely due to their greater tendency to present with symptoms. Furthermore, it is possible that asymptomatic vasospasms occurring in other regions have been missed. This sex-based predilection is consistent with previous reports indicating that women are more prone to experiencing vasospasm and are also at higher risk of severe outcomes [19]. Recent studies have highlighted the neuroprotective effects of progesterone in the context of early brain injury, which may partially explain the observed sex differences [20]. The factors contributing to the delayed onset remain unclear. However, if vasospasm is caused not by direct mechanical or hematoma stimulation, but by inflammatory cytokines - including IL-6 - induced by surgical stress, there is a possibility that a delayed onset of vasospasm may develop.

In the present case, vasospasm occurred in the M1 to M2 regions, which were not directly manipulated during the surgical procedure, as temporary clips were not used at any point. This may indicate that the underlying mechanism is potentially more associated with the influence of female hormones, such as estrogen, and inflammatory cytokines such as IL-6, rather than with direct endothelial cell injury resulting from surgical manipulation. In this case, the vasospasm occurred during hospitalization, which allowed for prompt intervention. However, if it had occurred after discharge, treatment or detection delays could have ensued, leading to severe neurological deficits. In fact, vasospasm after tumor resection can lead to a poor prognosis owing to its rarity and delays in diagnosis and treatment.

## Conclusions

Symptomatic cerebral vasospasm following the clipping of unruptured cerebral aneurysms is rare and represents a serious complication that can result in permanent neurological deficits if not promptly recognized and treated. The pathophysiology may involve endothelial damage caused by hematomas or direct surgical stimuli, as well as the involvement of sex hormones or inflammatory cytokines, such as IL-6, without direct stimulation. Key clinical points include that this condition is more prevalent and often more severe in women, frequently, and it has a higher likelihood of delayed onset compared with vasospasm associated with SAH. Further case accumulation and research are required to better understand the pathophysiology, identify risk factors, and establish optimal treatment protocols for this rare but potentially devastating complication.
